# Association of Complement Proteins with C Reactive Protein in Non-Obese Women with and Without Polycystic Ovary Syndrome

**DOI:** 10.3390/ijms26073008

**Published:** 2025-03-26

**Authors:** Alexandra E. Butler, Abu Saleh Md Moin, Hamna H. Begam, Sana Waris, Juberiya M. Azeez, Thozhukat Sathyapalan, Stephen L. Atkin, Edwina Brennan

**Affiliations:** 1 School of Postgraduate Studies & Research, Royal College of Surgeons in Ireland Bahrain, Adliya 15503, Bahrain; abutler@rcsi.com (A.E.B.); amoin@rcsi.com (A.S.M.M.); hbegam@rcsi.com (H.H.B.); swaris@rcsi.com (S.W.); jazeez@rcsi.com (J.M.A.); satkin@rcsi.com (S.L.A.); 2 Academic Endocrinology, Diabetes and Metabolism, Hull York Medical School, University of Hull, Hull HU6 7RU, UK; thozhukat.sathyapalan@hyms.ac.uk; 3 School of Medicine, Royal College of Surgeons in Ireland Bahrain, Adliya 15503, Bahrain

**Keywords:** polycystic ovary syndrome, C-reactive protein (CRP), inflammation, complement factors, BMI

## Abstract

Complement proteins are increased in polycystic ovary syndrome (PCOS), as are markers of inflammation, such as the C-reactive protein (CRP); however, both may be increased in obesity. We hypothesised that body mass index (BMI)-driven CRP would comparably associate with an increase in complement proteins when obesity was accounted for in non-obese women with and without PCOS. In a non-obese, non-insulin-resistant population without inflammation (24 with PCOS and 24 control women), plasma CRP was measured by immunoassay. Slow Off-rate Modified Aptamer (SOMA)-scan plasma proteomic analysis of the classical, lectin, and alternative pathway complement proteins was undertaken. BMI, insulin resistance, and CRP did not differ (*p* < 0.05) between the cohorts. The alternative pathway of the complement protein system was overexpressed in PCOS (*p* < 0.05). CRP correlated positively (*p* < 0.05) with alternate pathway parameters in women with and without PCOS for C3a, iC3b, Factor B, Factor H, and Factor I; in PCOS alone for C3, C3adesArg, and C3d; and in women without PCOS, for properdin. CRP did not correlate with lectin pathway C2 or MBL (*p* > 0.05). CRP correlated positively (*p* < 0.05) with C4 of the classical pathways in women with PCOS alone. Hyperandrogenemia did not correlate with CRP or complement in non-obese PCOS. BMI correlated positively with C3, C3adesArg, C3a, iC3b, Factor B, Factor H, and properdin: classical pathway proteins; C1q, C4, C5 and C5a in PCOS women; BMI only correlated negatively with C1q in non-PCOS women. Upregulation of complement proteins occur in non-obese PCOS, and CRP is positively associated with complement protein changes in both women with and without PCOS. This indicates that BMI induces changes in CRP that lead to changes in the complement pathways, particularly the alternate pathway, with increases in CRP (though still within the reference laboratory normal range) leading to upregulation of complement proteins in PCOS. This suggests an enhanced set point for CRP-induced complement protein dysregulation in PCOS.

## 1. Introduction

Polycystic ovary syndrome (PCOS) is associated with infertility and metabolic dysregulation in women, including an increased risk of type 2 diabetes, hypertension, and cardiovascular disease [1]. However, the precise pathophysiological mechanisms underlying these conditions in PCOS remain unclear. Obesity and insulin resistance, which contribute to systemic inflammation, are thought to play a central role in the pathophysiology of PCOS [2]. Additionally, oxidative stress, low vitamin D levels, and chronic inflammation have been identified as contributing factors [3,4]. Enhanced inflammation and oxidative stress in PCOS are linked to cellular alterations, including the dysregulation of heat shock proteins [5], changes in coagulation markers [6], and modifications in complement pathway proteins in which obesity and insulin resistance have been suggested to be the underlying mediators [7,8,9,10].

**Figure 1:**
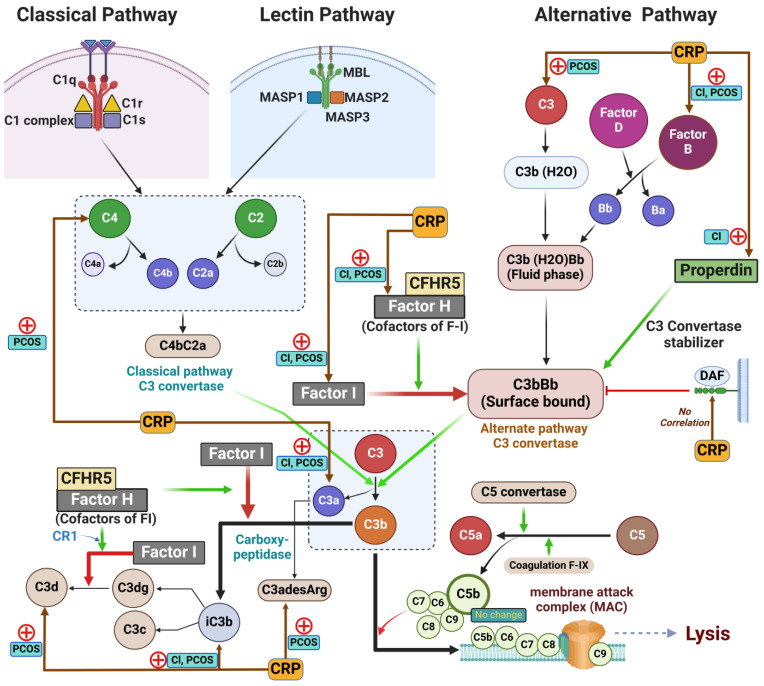
A schematic illustrating the initiating proteins of the classical (**left panel**), lectin (**middle panel**), and alternative (**right panel**) complement protein cascades. The green arrows in the illustration indicate the enzymatic activity or positive regulation, whereas the red arrows indicate the inhibition of pathways. The proteins that showed positive correlations with the C-reactive protein (CRP) were indicated by (+) signs. Cl, control; PCOS, polycystic ovary syndrome; C1–C9, complement protein component 1–9; MBL, Mannose-binding Lectin; MASP, Mannose-associated serine protease; DAF, Decay-accelerating factor; CFHR5, Complement factor H-related protein 5. Illustration was created using Biorender.com (accessed on 8 February 2025).

The complement protein system is an innate immune surveillance system composed of collaborative proteins that orchestrate pathogen opsonisation and inflammatory responses to combat infection and maintain homeostasis [11]. Whether activated through the classical, alternative, or lectin pathways, each lead to a common terminal pathway generating active C3 convertase [12] (Figure 1). Both the classical pathway, through C1 activation, and the lectin pathway, via mannose-binding lectin (MBL) activation, depend on the cleavage of C2 and C4, generating C3 convertase (C4b2b); whereas the alternative pathway, initiated through spontaneous hydrolysis of C3, relies on Factor B and Factor D to give rise to a distinct C3 convertase, C3bBb. C3 cleavage leads to subsequent C5 cleavage resulting in the recruitment of additional complement proteins which polymerise to form the membrane attack complex (MAC). This system is tightly regulated; for example, in the alternative pathway, properdin acts as a positive regulator stabilizing C3 convertase, while Factor H and Factor I inhibit its activity [11]. Studies have suggested that complement protein activation may contribute to the amplified inflammatory response observed in metabolic diseases, such as obesity, insulin resistance, diabetes [13,14], and cardiovascular disease [15], that are commonly associated with PCOS [1]. Enhanced expression of MBL has been reported in some women with PCOS, though this might vary depending on the population and associated metabolic complications [16]. In addition, both the classical and the alternative complement protein pathways have been implicated in PCOS, with elevated levels of C3, C4, properdin, Factor B, and Factor D reported in women with PCOS [7,10]. Notably, these elevations are often correlated with obesity and insulin resistance [7,8,9,10] (Figure 1).

C-reactive protein (CRP), a member of the pentraxin pentagonal superfamily, is primarily synthesised in hepatocytes in response to inflammation [17,18]. CRP expression is induced by interleukin (IL) 6 and Il-1β through promotor binding of its main transcriptional factors, members of the C/EBP family [19]. Acting as a nonspecific acute phase protein, with levels increasing from 0.8 to 500 μg/mL, CRP is used to monitor the severity of inflammation in a range of disease states [20,21]. CRP effectively binds to phosphocholine in a calcium dependent manner promoting phagocytosis of microbial cells as well as damaged and necrotic cells [22] and, once surface bound, activates the classical complement pathway proteins [23,24,25]. CRP limits inflammation through minimal generation of C5a and C5b-9 for formation of the MAC [26,27], which is achieved through recruitment of the negative complement regulator Factor H protein [28]. Therefore, the role of CRP is complex as a result of both its anti- and pro-inflammatory effects [29], and as a result of its interactions with various components of the complement protein system. Elevated CRP levels have been observed in women with PCOS, especially in those who are obese and insulin-resistant [30]. In their meta-analysis, Escobar-Morreale et al. [31] reported that CRP levels are nearly 100% higher in PCOS women than in control subjects. In their systematic review and meta-analysis, Aboeldalyl et al. [32] reported moderately elevated levels of circulating CRP in PCOS women independent of obesity when compared to control subjects. Also, circulating CRP levels are strongly associated with BMI, with evidence suggesting that CRP primarily serves as a marker of elevated adiposity rather than a causal factor in inflammation [33].

Analysing complement proteins in PCOS is challenging due to the strong association of PCOS with inflammation, obesity, and insulin resistance; whilst attempts can be made to adjust for these confounding factors in regression modeling, this may lead to overcorrection, thus obscuring the underlying pathogenesis. To address this, this study compared non-obese, non-insulin-resistant PCOS subjects who did not exhibit increased systemic inflammation (as adjudged by CRP within normal laboratory limits) with age and BMI matched controls. Our hypothesis was, that in such a matched population where systemic inflammation was absent, CRP levels would not be related to complement factors, and that these factors would not differ significantly between women with or without PCOS.

## 2. Results

### 2.1. Demographics and Biochemical Measurements

Baseline data for the 24 non-obese, non-insulin resistant PCOS and controls are shown in Table 1. Age and BMI were matched, the women with PCOS were not insulin resistant, and CRP did not differ between the groups.

### 2.2. Complement Protein Activation in Non-Obese Women with and Without PCOS [9,34]

The results of the complement protein factors are shown in Table 2 for the non-obese women with PCOS and their respective control subjects. There was no difference in the classical pathway levels of C1q and C1r between non-obese PCOS women and control women, nor did C4 differ between the groups. There was no difference in the levels of C2 or MBL proteins involved in the activation of the lectin pathway between non-obese PCOS and control women. C3 levels were higher in non-obese PCOS compared to controls (65,878 ± 26,872 vs. 45,742 ± 18,189 RFU of C3, *p* = 0.002); the anaphylatoxin fragment of C3 cleavage, C3a, as well as its sequential cleavage product, C3adesArg, were elevated in non-obese PCOS compared to controls (534 ± 204 vs. 415 ± 101 RFU, *p* = 0.007, and 152,050 ± 32,483 vs. 121,110 ± 45,753 RFU, *p* = 0.004, respectively). The binding fragment of C3 cleavage, C3b, did not differ; however, the negative regulator of C3b, Factor I, was higher in non-obese PCOS compared to controls (44,861 ± 6786 vs. 39,960 ± 7356, *p* = 0.01), as were the levels of Factor I degradation products of C3b cleavage, iC3b and C3d (7148 ± 2127 vs. 5991 ± 1425, *p* < 0.02, and 10,427 ± 3675 vs. 8207 ± 3261, *p* = 0.02, non-obese PCOS vs. control, respectively). There was no difference in levels of negative regulators Factor H, complement factor H-related protein 5 (CFHR5), or decay-accelerating factor (DAF) in non-obese PCOS compared to controls (Table 2). Among the positive regulators of the alternative pathway, Factor B and Factor D did not differ in non-obese PCOS compared to controls; however, properdin levels were higher in non-obese PCOS compared to controls (152,592 ± 42,743 vs. 117,488 ± 50,041 RFU, *p* = 0.006). Of the terminal pathway proteins, C5a levels were higher in non-obese PCOS compared to controls (14,729 ± 5811 vs. 11,343 ± 4953, *p* = 0.02), and there was no difference in C5, C5b, or C6 complex levels.

### 2.3. Correlations of CRP and Hyperandrogenemia with BMI

**Figure 2:**
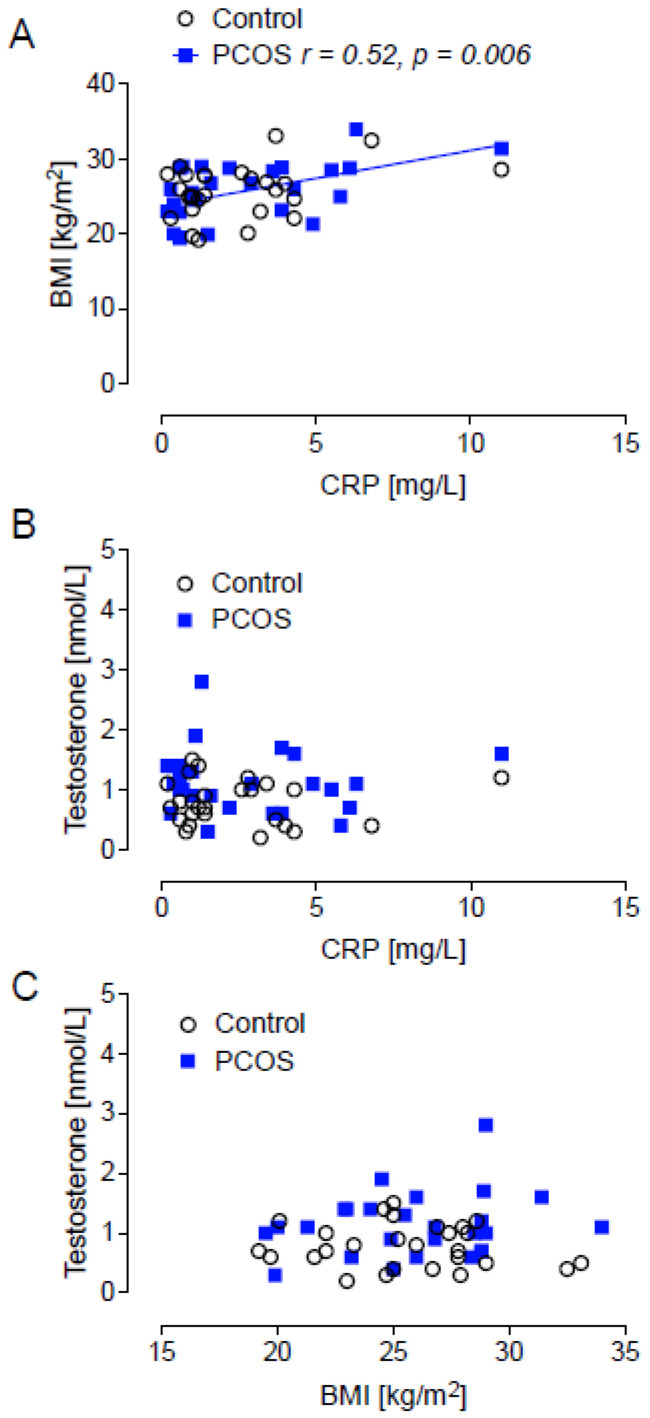
Correlations of BMI with CRP (**A**); testosterone with CRP (**B**); testosterone and BMI (**C**). There was a positive correlation of BMI with CRP in the PCOS cohort only, whilst no correlation was seen for testosterone with CRP or testosterone with BMI in either cohort.

BMI was positively and moderately correlated with CRP in PCOS (*r* = 0.52, *p* < 0.006), but not in the control population (Figure 2A). Testosterone did not correlate with BMI, nor with CRP (Figure 2B,C).

### 2.4. Correlations Between CRP and Complement Proteins in Non-Obese Women with and Without PCOS

**Figure 3:**
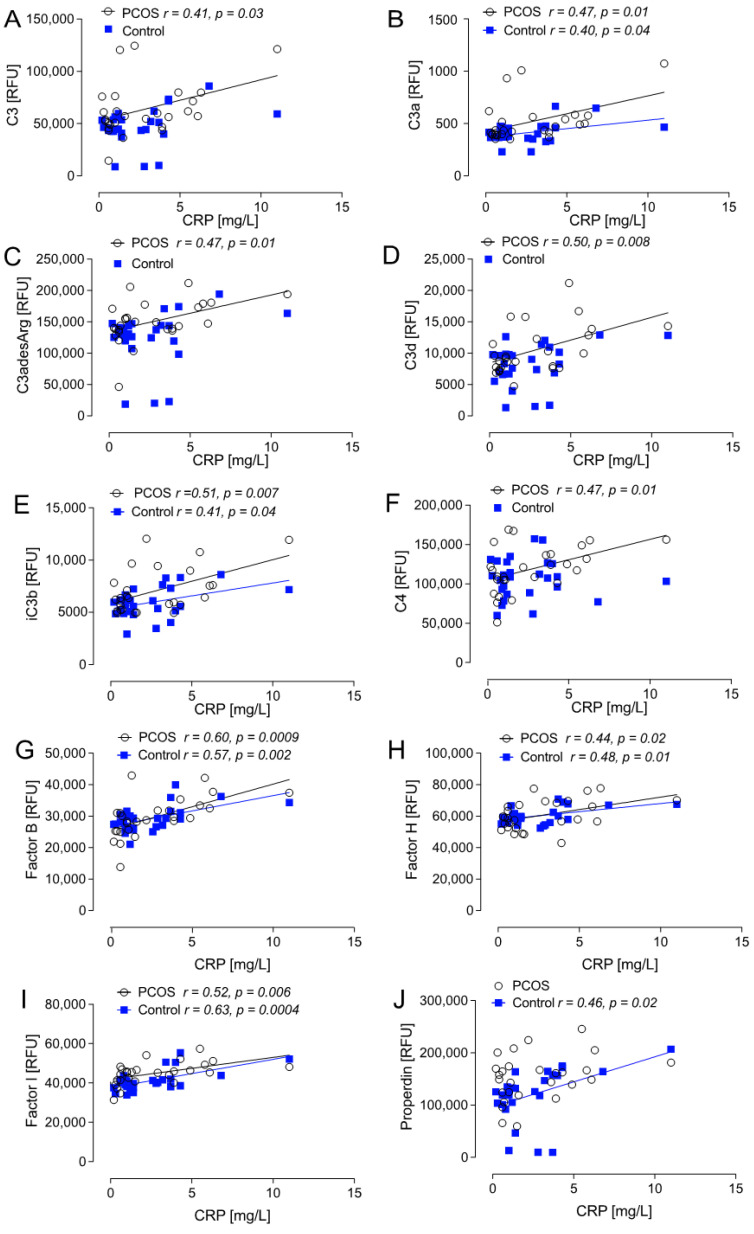
Correlations of complement proteins with CRP in women with non-obese PCOS (*n* = 24) and weight matched controls (*n* = 24): C3 (**A**); C3a (**B**); C3adesArg (**C**); C3d (**D**); iC3b (**E**); C4 (**F**); Factor B (**G**); Factor H (**H**); Factor I (**I**); and Properdin (**J**). Relative Fluorescent Units (RFU).

Correlations between the complement pathway proteins and CRP are shown in Figure 3. In non-obese PCOS women alone, CRP positively correlated with C4 (*r =* 0.47, *p* = 0.01). CRP did not correlate with levels of C1q, C1r, C4a, or C4b. CRP did not correlate with C2 or with MBL in non-obese women with and without PCOS. In non-obese PCOS women alone, CRP positively correlated with complement proteins C3 (*r =* 0.41, *p* = 0.03), C3adesArg (*r =* 0.47, *p* = 0.01), and C3d (*r =* 0.5, *p* = 0.008). In non-obese women both with and without PCOS, CRP positively correlated with the complement proteins C3a (*r =* 0.47, *p* = 0.01 PCOS: *r =* 0.40, *p* = 0.04 controls) and iC3b (*r =* 0.51, *p* = 0.007 PCOS: *r =* 0.41, *p* = 0.04 controls). In non-obese women without PCOS, CRP positively correlated with the positive regulator of the AP, properdin (*r* = 0.46, *p* = 0.02). In non-obese women both with and without PCOS, CRP positively correlated with the positive regulator of the AP, Factor B (*r =* 0.6, *p* = 0.0009 PCOS: *r =* 0.57, *p* = 0.002 controls) and the negative regulators of the AP, Factor I (*r =* 0.52, *p* = 0.006 PCOS: *r =* 0.63, *p* = 0.0004 controls) and Factor H (*r =* 0.44, *p* = 0.02 PCOS: *r =* 0.48, *p* = 0.01). There were no correlations between CRP and the negative regulators CFHR5 or DAF. There were no correlations observed for CRP with C5, C5a, or C5b-6 complex in non-obese women with or without PCOS.

### 2.5. Correlations of Complement Activation Related Proteins with Hyperandrogenemia, and BMI [34]

Hyperandrogenemia did not correlate with any complement protein factors in non-obese PCOS. BMI correlated positively in non-obese PCOS women as follows: with alternative pathway complement proteins, C3 (*r* = 0.56, *p* = 0.002), C3adesArg (*r* = 0.5, *p* = 0.005), C3a (*r* = 0.53, *p* = 0.003), iC3b (*r* = 0.46, *p* = 0.01), Factor B (*r* = 0.66, *p* < 0.0001), Factor H (*r* = 0.58, *p* < 0.0001), and properdin (*r* = 0.58, *p* = 0.001); with classical pathway proteins, C1q (*r* = 0.54, *p* = 0.02), C4 (*r* = 0.46, *p* = 0.01), C5 (*r* = 0.39, *p* = 0.04), and C5a (*r* = 0.61, *p* < 0.0004). In non-obese women without PCOS, BMI only correlated negatively with C1q (*r =* −0.33, *p* = 0.04). No correlations were observed between lectin pathway complement proteins C2 or MBL.

## 3. Discussion

The results of this study show that, in the PCOS cohort, levels of alternative pathway complement proteins and regulators were increased compared to age and BMI matched controls, while there was no difference in the classical or lectin pathway parameters, and minimal activation of the terminal proteins. Furthermore, BMI-related changes in CRP correlated positively with complement protein changes in both women with and without PCOS, changes that were not related to hyperandrogenemia in the PCOS cohort.

Although there are a limited number of studies that have examined complement activation in PCOS, altered complement activation proteins have been reported as follows: increased [7,8,10,35,36] and similar [37,38,39,40] C3 levels; increased [7,35,36,37] and similar [10] C3adesArg levels; increased [10] and similar [7,40] properdin levels; increased [41] and similar [7,10,40] Factor D levels; increased [10] and similar [7,40] Factor H and Factor B levels; similar [7,10,40] C4 and C5 levels; increased [10] and similar [7,40] C5a levels; increased [40] and similar [10] C4b levels; increased [10] and similar [40] Factor I and C2 levels; increased [40] C3b/iC3b levels; increased [10] iC3b and C3d levels. However, comparisons with this study and published studies are difficult, given that most published studies on complement activation proteins in PCOS are confounded by obesity and insulin resistance; whereas, here, those factors, as well as systemic inflammation, were accounted for.

CRP-mediated complement activation is most effective at C1 of the classical pathway and where it converges with the lectin pathway at C2 and C4 [28]; it has been reported that complement activation by the CRP only occurs with activated CRPs [42]. In this study, where CRP levels did not differ between PCOS and control women, there was no difference in the classical pathway activation parameters (C1q, C1r), in the lectin pathway parameters (MBL, C2), or in combined parameters (C4a, C4b), and none correlated with CRP. Only C1q of the classical pathway correlated with BMI, positively in PCOS and negatively in controls. Based on the results here, and those of previous studies [10,40], the classical and lectin pathway activation parameters are unaltered in PCOS regardless of commonly associated pathophysiological states, such as obesity, insulin resistance and systemic inflammation. The lack of activation of the classical pathway reported here is in accord with that of Basile et al. [43], who reported reduced levels of immunoglobulins and lower classical pathway activation, measured through CH50 analysis, in non-obese PCOS. Although not significant (*p* = 0.05), C4 was higher in PCOS compared to controls, consistent with other reports [40], and positively correlated with CRP and BMI in PCOS alone. However, other studies have reported no difference in C4 levels when BMI was accounted for [7] or in the presence of inflammation [10]. Given that the cleavage products (C4a, C4b) were no different, this suggests that C4 activation does not occur in PCOS. Instead, C4 may be linked to risk factors associated with PCOS [1], such as increased adipose tissue and cardiovascular affects [44].

Although moderate, CRP has the ability to activate C3 [28], resulting in cleavage to C3a and C3b. C3a binds to its G protein-coupled C3a receptor, inducing a localised inflammatory response [12], and is subsequently converted by carboxypeptidase action to C3adesaArg, a non-inflammatory triglyceride storage regulator of adipose tissue [45]. Here, C3 and C3adesArg, in PCOS alone, and C3a, in both PCOS and controls, positively correlated to CRP, while all positively correlated to BMI in PCOS alone. C3 strongly associates with inflammation, adipose tissue, and cardiovascular risk factors [44], and both C3a and C3adesaArg are highly associated with obesity, insulin resistance, and lipid metabolism disorders [46]. The increased levels reported here are somewhat consistent with previous studies where insulin resistance and inflammation were not accounted for [8,10,35,36], but BMI was accounted for [7], suggesting that, while circulating levels of C3, C3a, and C3adesArg are related to obesity, insulin resistance, and inflammation, the levels are also independent and an inherent feature of PCOS, indicating an enhanced set point for CRP and BMI-induced complement changes in PCOS.

In the alternative pathway, the larger cleavage product of C3, C3b, complexes with Factor B, which is subsequently activated by Factor D into active C3 convertase, creating a positive feedback loop and amplification of the complement effect [47]. The C3bBb convertase, however, has a relatively short half-life (approximately 90 s) [48]. Properdin, through binding of C3b, as well as Factor B and Bb [49], acts as a positive regulator of the alternative pathway, stabilizing C3bBb and increasing its half-life 5–10 fold [50]. Here, properdin was increased in PCOS, and positively correlated with BMI in PCOS and with CRP in controls alone. There are conflicting reports with regards to properdin levels in PCOS, with some reporting increased levels [10], while others report no difference [7,40]. Nevertheless, it appears that, in this PCOS cohort, properdin stabilisation is ineffective, given that there were no differences in C3b levels. Although no correlations with CRP were observed for properdin and C3b in PCOS, in the acute phase response of inflammation, circulating CRP levels dramatically increased up to 500-fold while properdin levels decreased [18]. In the inflammatory environment, the native conformation of CRP dissociates to form a monomeric modified CRP (mCRP) [51]. mCRP is capable of binding ligands not bound by native CRP in human cells, including C3b [52] and properdin [53], thereby inhibiting C3 activation and MAC formation. Whether the ineffective stabilisation of the AP reported here is due to interaction with mCRP rather than CRP requires further investigation.

Regarding the positive regulators Factor B and Factor D, there was no difference in the levels in PCOS or controls. This contrasts with the study by Gursoy Calan et al. [41], who reported increased odds of PCOS with increased Factor D levels independent of BMI, insulin resistance, free testosterone, or CRP levels, although all positively correlated with Factor D. Here, Factor B positively correlated to CRP but not to Factor D. Factor B positively correlated to BMI, which is consistent with increased levels in obese PCOS [10], but not in BMI adjusted models [7]. The lack of correlation between Factor D and CRP here is consistent with human serum studies where Factor D levels were unaffected by complement protein consumption by CRP [54].

Conversely, the subsequent and terminal cleavage products of C3b, iC3b, and C3d, respectively, were increased in PCOS. In addition, both positively correlated with CRP and BMI, iC3b correlated with CRP in PCOS and controls, C3d correlated with CRP in PCOS alone, and both correlated with BMI in PCOS alone. We also observed an increase in Factor I in PCOS, which positively correlated with CRP in PCOS and controls. Cleavage of C3b to iC3b and C3d by Factor I inhibits alternative pathway initiation and terminal complement protein cascade activation [55]. Factor H, which was similar in PCOS and controls, positively correlated with CRP in PCOS and controls, and with BMI in PCOS alone. Factor H is a cofactor for Factor I inactivation of C3b and competes with Factor B for alternative pathway C3 convertase activation [56]. Surface bound CRP recruits Factor H to limit the inflammatory response to opsonisation without generation of the MAC [28]. Reports indicate that this is supported by Factor H interaction with C3d [57], which is involved in amplifying B-cell immunity [58]. The dysregulation of these alternative pathway proteins and regulators suggests enhanced suppression of alternative pathway activation in PCOS at sites of tissue damage and local inflammation. Given that increased levels of iC3b, C3d, and Factor I were reported in obese PCOS with inflammation [10], this dysregulation appears inherent to PCOS. Furthermore, the association with BMI and CRP suggests exaggerated effects when obesity and inflammation are present.

DAF and CFHR5 are both negative regulators of the alternative complement pathway proteins. DAF is a membrane cofactor for Factor I-mediated proteolysis of C4b and C3b [11], competes with Factor B for binding to C3b, and can dissociate the Bf fragment from active convertase [12]. CFHR5 acts as a cofactor for Factor I and DAF to inhibit C3b [59]. CFHR5 has been demonstrated to have a binding site for CRP, and CRP recruitment of CFHR5 at sites of tissue damage may inhibit alternative pathway C3 convertase activity [60]. In animal models, DAF has been found to attenuate the effects of CRP-induced complement tissue damage [61]. In this study, there were no differences in DAF and CFHR5 levels, and no correlations were found with CRP or BMI. This suggests that the DAF and CFHR5 are not involved in PCOS or PCOS associated effects and that the previously reported increased levels of CFHR5 [10] were BMI driven.

In the terminal pathway of complement activation, C5 convertase cleaves C5 to C5a and C5b, the latter giving rise to the MAC [12]. Generation of C5a, a potent anaphylatoxin, is reported to stimulate properdin release for complement activation amplification [11]. Conversely, CRP has been found to inhibit the C5a-induced chemotaxis of neutrophils in vitro [62], although the exact mechanism is unknown. In this study, only C5a levels were increased in PCOS alone. C5 and C5a both positively correlated to BMI, but no correlations with CRP were observed. Consistent with other studies [7,10,40], there was no difference in C5 levels, suggesting that C5 activation does not occur in PCOS regardless of insulin resistance or inflammation, although levels may be impacted by obesity, given its correlation with BMI. There are conflicting reports in the literature with regards to C5a levels in PCOS, with some studies reporting increased levels [10], as reported here, while others reporting no difference [7,40]. If increased levels of C5a are inherent to PCOS, there appears to be incomplete terminal complement activation, given the lack of difference in C5 and C5b levels, the latter of which is underreported in the literature. Given C5 cleavage can occur through phagocytic cell action [63] and through interaction with thrombin [64], additional studies are required to confirm the role of C5a in PCOS.

While adjustment of confounding variables in regression modeling can be very powerful, such efforts in PCOS may mask the pathogenesis of the disorder; this was avoided here by the study design of non-obese, non-insulin-resistant PCOS subjects who did not exhibit increased systemic inflammation with age and BMI matched controls. Given the sample size (*n* = 24), it is possible that Type II statistical errors (false negatives) may have occurred, as smaller samples inherently reduce the likelihood of detecting true differences. However, a power analysis was undertaken based on the best available literature, and this study was deemed to have adequate power to detect clinically meaningful differences. The absence of significant findings for some parameters may reflect a true lack of difference, rather than being solely due to insufficient power. Nonetheless, we acknowledge that the superiority framework used in this study, while appropriate for detecting differences, is not optimal for conclusively establishing equivalence. Therefore, to definitively determine whether the observed lack of significant differences truly reflects equivalence between the groups, future research employing non-inferiority or equivalence designs in larger, more diverse cohorts is warranted. Such studies would provide more robust insights into potential subtle differences and enhance the generalisability of these findings. As all participants in this study were Caucasian, comparisons with other races or ethnicities may not be possible given such impacts on PCOS phenotypes [65], which were also not addressed here. In this study, all of the PCOS subjects had anovulatory infertility, but half were phenotype B (irregular menses with hyperandrogenism) and half were phenotype C (irregular menses and polycystic ovaries on transvaginal scanning); there were too few subjects, and less than the power analysis would allow, to perform a subgroup analysis. Therefore, future larger studies need to determine if the four PCOS phenotypes affect individual parameter expression. Only protein levels were measured, and future studies should also utilise functional complement activity assays as the elevated complement protein levels reported here may not have enhanced functional activity. Insulin resistance was determined by homeostasis model assessment (HOMA-IR), and whilst a more sensitive method based on the oral glucose tolerance test would have been preferable, this was only performed for the PCOS subjects and not for controls. 

In conclusion, upregulation of complement protein system factors occurs in non-obese, non-insulin resistant PCOS without systemic inflammation. CRP is associated positively with complement changes in both women with and without PCOS, indicating that BMI induces changes in CRP that lead to changes in the complement pathways, particularly the alternate pathway, that are not associated with hyperandrogenemia. Increases in CRP within the reference laboratory range may lead to an exaggeration of complement protein levels in PCOS, suggesting an enhanced set point for CRP-induced complement changes in PCOS.

## 4. Materials and Methods

We determined plasma complement pathway protein levels in sequential women with PCOS (*n* = 24) and control (*n* = 24) women attending the Hull IVF clinic [66]. Control women were age and BMI matched to the women with PCOS. All subject inclusion criteria were age 20–40 years, BMI ≤ 30. Control women’s reason for in vitro fertilisation was either unexplained infertility or male factor infertility. All subjects fulfilling the inclusion and exclusion criteria who were attending for a routine clinical mock embryo transfer procedure were approached about inclusion in this study and signed written informed consent. No woman had a previous history of recent infection or autoimmune disease. Study approval was granted by The Yorkshire and The Humber NRES ethical committee, UK (approval number 02/03/043).

All women were Caucasian, were from the same demographic area and with low socioeconomic background. The diagnosis of PCOS was based on at least two of the three diagnostic criteria of the Rotterdam consensus, as detailed previously [67]; namely, clinical and biochemical evidence of hyperandrogenism (Ferriman–Gallwey score > 8; free androgen index > 4), oligomenorrhea or amenorrhoea, and polycystic ovaries on transvaginal ultrasound. Nonclassical 21-hydroxylase deficiency, hyperprolactinemia, Cushing’s disease, and androgen secreting tumors were excluded by appropriate tests. The baseline study measurements have been described in detail previously [68], and the demographic data for the PCOS and control women is shown in Table 1. All women with PCOS had an oral glucose tolerance test to exclude prediabetes and diabetes; however, it is possible that there were some subjects with insulin resistance to a glucose load that would have not been detected. All the control women recruited by advertisement had regular periods, no clinical or biochemical hyperandrogenism, no polycystic ovaries on ultrasound, no significant background medical history, and none of them were on any medications, including oral contraceptive pills or over-the-counter medications.

### 4.1. Sample Analysis

Fasting blood samples were centrifuged at 3500× *g* for 15 min, placed into aliquots and frozen at −80 °C until analysis. The blood was analysed for CRP, sex hormone binding globulin (SHBG), insulin (DPC Immulite 200 analyser, Euro/DPC, Llanberis, UK), and plasma glucose (Synchron LX20 analyser, Beckman Coulter, Inc., High Wycombe, UK). Free androgen index (FAI) was calculated by dividing the total testosterone by SHBG and then multiplying by one hundred. Insulin resistance (IR) was calculated using the HOMA-IR with a cut-off value of 2.1. Serum testosterone was quantified using isotope-dilution liquid chromatography tandem mass spectrometry (LC-MS/MS) (Architect analyzer, Abbott Laboratories, Maidenhead, UK) [66]. Circulating levels of complement pathway proteins were determined by Slow Off-rate Modified Aptamer (SOMA)-scan plasma protein measurement, the details of which have been previously reported [69]. Normalisation of raw intensities, hybridisation, median signal, and calibration signal were performed based on the standard samples included on each plate, as previously described [70]. We used version 3.1 of the SomaScan Assay, specifically targeting those proteins involved in the classical and alternative complement pathways in the SomaScan panel, some of which have been reported by others previously [7]. These included the following complement system proteins for the classical, alternative, and lectin pathways: C1q, C1r, C2, C3, C3a, iC3b, C3b, C3d, C3adesArg, C4, C4a, C4b, C5, C5a, C5b-6 complex, C8, properdin, Factor B, Factor D, Factor H, Factor I, MBL, DAF, and CFHR5 (Figure 1, Table 2).

### 4.2. Statistics

A power analysis (nQuery version 9, Statsols, Boston, MA, USA) was undertaken for the C3 protein previously reported to be different in PCOS [7]. For 80% power and an alpha of 0.05 with a common standard deviation of 0.37, the number of subjects required was 23. Data trends were visually and statistically evaluated for normality. Independent *t*-tests were applied on normally distributed data, while non-parametric tests (Mann–Whitney U) were applied on data that violated the assumptions of normality when tested using the Kolmogorov–Smirnov Test. Correlation analyses were carried using Pearson coefficient. All analyses were performed using R version 4.0.0 (R Foundation for Statistical Computing, Vienna, Austria. URL https://www.R-project.org/ (accessed on 24 November 2024).

## Figures and Tables

**Table 1:** Demographics, baseline, hormonal, and metabolic parameters of the PCOS subjects and controls (mean ± SD).

	PCOS (*n* = 24)	Control (*n* = 24)
Age (years)	31 ± 6.4	32.5 ± 4.1
BMI (kg/m^2^)	25.9 ± 1.8	24.8 ± 1.1
Insulin (IU/mL)	8.1 ± 4.7	7.7 ± 4.0
HOMA-IR	1.9 ± 1.6	1.8 ± 1.0
Testosterone (nmol/L)	1.4 ± 0.8	0.7 ± 0.4 ***
SHBG (nmol/L)	71.7 ± 62.2	104 ± 80
FAI	4.1 ± 2.9	1.3 ± 0.5 **
CRP (mg L^−1^)	2.8 ± 2.6	2.3 ± 2.34
AMH (ng/mL)	57 ± 14	24 ± 13 **

BMI, Body Mass Index; HOMA-IR, Homeostasis model assessment of insulin resistance; FAI, Free androgen index; CRP, C reactive protein; SHBG, sex hormone binding globulin; AMH, Anti-Müllerian hormone. ** *p* < 0.01, *** *p* < 0.001.

**Table 2:** Complement proteins in non-obese, non-insulin-resistant patients with polycystic ovary syndrome (PCOS) versus their BMI-matched controls. Data presented as Mean ± 1 Standard Deviation of Relative Fluorescent Units (RFU).

			PCOS	Control	*p* Value
Alternative pathway	Proteins	C3	65,878 (26,872)	45,742 (18,189)	0.002
		C3a	534 (204)	415 (101)	0.007
		C3adesArg	152,050 (32,483)	121,110 (45,753)	0.004
		C3b	50,982 (28,296)	46,250 (39,450)	0.6
		iC3b	7148 (2127)	5991 (1425)	0.02
		C3d	10,427 (3675)	8207 (3261)	0.02
	Negative regulators	Factor I	44,861 (6786)	39,960 (7356)	0.01
		Factor H	60,898 (9191)	59,289 (6016)	0.43
		CFHR5	1454 (399)	1677 (1416)	0.42
		DAF (CD55)	15,182 (3220)	13,886 (3339)	0.14
	Positive regulators	Factor B	30,257 (6541)	28,172 (5791)	0.20
		Factor D	733 (99)	693 (150)	0.24
		Properdin	152,592 (42,743)	117,488 (50,041)	0.006
Classical pathway	Proteins	C1q	35,146 (9269)	35,294 (7886)	0.95
		C1r	3203 (886)	3740 (3992)	0.48
		C4	119,057 (28,429)	104,245 (28,069)	0.05
		C4a	71,549 (9802)	73,037 (2258)	0.43
		C4b	349 (186)	335 (202)	0.78
Lectin pathway	Proteins	C2	2878 (319)	2874 (228)	0.96
		MBL	12,058 (5835)	13,322 (7385)	0.47
Terminal pathway	Proteins	C5	7267 (1007)	6881 (1063)	0.16
		C5a	14,729 (5811)	11,343 (4953)	0.02
		C5b, 6 Complex	521 (62)	502 (62)	0.27

## Data Availability

All the data for this study will be made available upon reasonable request to the corresponding author.

## Abbreviations

The following abbreviations are used in this manuscript:

PCOS	Polycystic ovary syndrome
CRP	C-reactive protein
BMI	Body mass index
MBL	Mannose-binding lectin
MAC	Membrane attack complex
SOMA	Slow Off-rate Modified Aptamer
HOMA-IR	Homeostasis model assessment of insulin resistance
SHBG	Sex hormone binding globulin
FAI	Free androgen index
AMH	Anti-Müllerian hormone
DAF	Decay-accelerating factor
CFHR5	Complement factor H-related protein 5
